# The Effect of a Parent Advocacy Intervention on Service Access for Autistic Youth: The Moderating Role of Baseline Advocacy Ability

**DOI:** 10.1002/aur.70357

**Published:** 2026-08-26

**Authors:** Carly Moser, Leann Smith DaWalt, Meghan M. Burke, Julie Lounds Taylor

**Affiliations:** ^1^ Vanderbilt University Medical Center Nashville Tennessee USA; ^2^ University of Wisconsin–Madison Madison Wisconsin USA; ^3^ Vanderbilt University Nashville Tennessee USA

**Keywords:** adult disability services, autism, parent advocacy, transition to adulthood, treatment moderators

## Abstract

Autistic youth face significant challenges accessing adult disability services as they transition out of school‐based services. Advocating for Supports to Improve Service Transitions (ASSIST), a group‐based program, was developed to address this issue by teaching parents about the service system and how to advocate for services for their youth. Through a randomized controlled trial, previous findings showed that ASSIST improved parent advocacy ability and increased access to government programs for youth who had exited high school. To identify which families may benefit most from ASSIST, the current study used a baseline target moderation framework to examine whether parents' baseline levels of advocacy ability (i.e., knowledge of adult services, perceived advocacy skills, and empowerment) modified the effect of intervention modality (ASSIST vs. written materials) on the primary outcome. The primary outcome for this study was having one or more service needs met at the 6‐ or 12‐month follow‐up. Participants included 138 parents of transition‐aged autistic youth who received the ASSIST program or comprehensive written materials on disability services. We found that parents taking ASSIST who reported high levels of perceived advocacy skills and empowerment at baseline were more likely to have a service need met compared to those who received written materials. These findings help clarify which families are most likely to benefit from a parent advocacy program and underscore the importance of parental empowerment and perceived advocacy skills in securing needed services for their autistic youth.

## Summary

After high school, services can be hard to access for autistic youth. To help, we developed a parent advocacy training program called Advocating for Supports to Improve Service Transitions (ASSIST). In this study, we wanted to understand which families may benefit the most from ASSIST, which we defined as receiving at least one service that they needed. We found that families in ASSIST were more likely to receive a needed service when parents came into the study with high levels of perceived advocacy skills and empowerment.

## 1 Introduction

In the United States, the Individuals with Disabilities Education Act (IDEA) ensures that youth with disabilities, including those with autism, are eligible for mandated services through the school system (IDEA 2004). However, after leaving the school system, autistic youth encounter an adult disability service system that is both underfunded and difficult to navigate (Anderson and Butt 2018; Marsack‐Topolewski and Weisz 2020). Even when youth qualify for services, multiple factors, such as inadequate provider availability or direct support staff shortages, often limit actual service receipt (Dawson et al. 2024). Improving access to adult disability services is critical for supporting autistic individuals, as these services help address needs in areas such as independent living, community engagement, and employment, and are a high priority for stakeholders in the autism community (e.g., autistic adults, family members, professionals; Roche et al. 2021). Parents' ability to advocate for services, including knowledge of adult disability services, perceived advocacy skills, and empowerment, may be particularly important facilitators of service access during the transition to adulthood. Burgeoning evidence supports this premise (Anderson and Butt 2018; Casagrande and Ingersoll 2017; Lee et al. 2022); a study by Lee et al. (2022) found that the number of advocacy activities parents engaged in was strongly associated with the number of services their transition‐aged autistic youth received.

Given the important role of parent advocacy, interventions have been developed to increase parents' ability to advocate for services on behalf of their children with disabilities (Burke, Goldman, et al. 2016; Magaña et al. 2017; Taylor et al. 2017, 2026). However, little is known about the characteristics of families who are most likely to benefit from these interventions and those who may require more targeted support. The present study begins to address this gap by examining whether parents' initial levels of advocacy ability impact the effectiveness of a parent advocacy program in improving service access.

### 1.1 Indicators of Advocacy Ability

#### 1.1.1 Knowledge of Adult Disability Services

To effectively advocate for adult disability services, an understanding of the disability service system is essential; however, parents of autistic youth report a lack of knowledge in this area (Chen et al. 2019; Hoffman and Kirby 2022). In a qualitative study involving 39 parents of autistic transition‐aged youth, participants indicated a lack of awareness regarding where to find services and what kinds of services were available. Parents who were generally aware of specific services, such as Vocational Rehabilitation, needed more clarity on the role these programs would play in supporting their youth (Hoffman and Kirby 2022). For autistic youth and their families, understanding eligibility criteria for programs is crucial, as requirements and priorities for service receipt based on functional abilities vary widely across service systems and states. Other complicated circumstances for youth arise due to income eligibility requirements. For instance, in some states, employees with disabilities cannot qualify for Medicaid health insurance because of their earnings, while other states provide a “Medicaid Buy‐In” program, allowing employees with disabilities to keep their health care coverage (Centers for Medicare & Medicaid Services n.d.). Understanding available programs and their unique eligibility requirements is essential to being able to identify appropriate services, effectively advocate when families encounter barriers, and guarantee ongoing coverage.

#### 1.1.2 Perceived Advocacy Skills

In addition to understanding the service system, parents must possess the skills needed to effectively communicate about their child's needs and feel comfortable engaging in these efforts (Burke, Goldman, et al. 2016). Developing these advocacy skills enables parents to persist in their efforts to access and improve services for their child, even if they are faced with barriers (Burke, Patton, and Lee 2016; Kildahl et al. 2026; Wang et al. 2004). For instance, in a qualitative study of eight parents of young adults with complex support needs, parents described using their advocacy skills to voice their concerns about service quality; when this led to increased conflict with providers, they continued to advocate to ensure their young adult received the support they needed (Kildahl et al. 2026).

#### 1.1.3 Empowerment

Finally, parents need to feel empowered to follow through with the knowledge and skills they have accumulated to gain access to services for their autistic youth. Family empowerment can be defined as the attitude, knowledge, and behaviors that enable individuals to gain more control over meeting their child's needs and can occur at different levels: within the family, the service system, and the community or political system (Koren et al. 1992). Empowerment is associated with increased participation in advocacy activities (Li et al. 2024) and has been linked to greater satisfaction and involvement with services (Curtis and Singh 1996; Resendez et al. 2000). Taken together, to advocate effectively, parents need to (1) have knowledge of the disability service system, (2) feel confident they have the skills to advocate, and (3) feel empowered to speak on behalf of their autistic young adults.

### 1.2 Effectiveness of Parent Advocacy Programs

Given the link between parent advocacy ability (i.e., knowledge, perceived advocacy skills, and empowerment) and service access, interventions have been developed to target advocacy ability as a way to improve service access. Advocacy programs for parents of school‐aged children with disabilities have led to improved advocacy ability, including increased special education knowledge, perceived advocacy skills, and empowerment (for a review, see Rios and Burke 2021). Based on these positive findings in families of school‐aged children, our team developed a parent advocacy program focused on increasing access to adult disability services, called Advocating for Supports to Improve Service Transitions (ASSIST). ASSIST is a 12‐session group‐based program that aims to improve service access for autistic youth transitioning to adulthood by teaching parents about different aspects of the adult disability service system and how to effectively advocate for services and supports. ASSIST was tested through a randomized controlled trial (RCT) design with parent advocacy ability (i.e., knowledge, perceived advocacy skills, and empowerment) as the mechanism of action hypothesized to increase service access for autistic youth (Taylor et al. 2022).

Results from the RCT suggest that, for some outcomes, parents who participated in ASSIST (i.e., the treatment group) experienced greater benefit than those who received comprehensive information about adult services in written form (i.e., the comparison group). For other outcomes, parents seemed to benefit from both conditions. In terms of advocacy ability, for example, those who participated in ASSIST showed greater gains in knowledge about adult services and perceived advocacy skills than the comparison group, but the two groups experienced comparable gains in empowerment (Taylor et al. 2023). In terms of service access, 6 months following ASSIST, participants in the treatment (versus comparison) group reported receiving more government programs (Taylor et al. 2026). However, this improvement was evident only among families of autistic youth who had already left high school at the time that their parents participated in ASSIST. A considerable number of participants in the treatment and comparison groups reported receiving more government programs 6 months after the intervention, suggesting that—at least for some parents—receiving comprehensive written materials about adult services may be sufficient to improve service access (Taylor et al. 2026). These findings align with those from Young et al. (2016), who found that providing a brochure on transition services for students with disabilities can improve knowledge of disability services, although not as much as additional parent training. Delivering a 12‐week advocacy program is more resource‐intensive than providing written materials, both for organizations delivering the program and for parents who have to dedicate time to attend. Therefore, to inform efficient targeting of resources, it is important to understand the characteristics of families who are most likely to benefit from each of these intervention modalities (i.e., ASSIST versus written materials).

### 1.3 The Present Study

In the present study, we focused on baseline parent advocacy ability as a characteristic that may impact the effect of intervention modality (i.e., ASSIST class versus written materials) on treatment outcomes. Informed by the baseline target moderation framework (Howe 2019; Howe and Leijten 2023), we hypothesized that initial levels of advocacy ability (i.e., knowledge, perceived advocacy skills, and empowerment) would modify the effect of the intervention modality (i.e., ASSIST class versus written materials) on the primary outcome (see the hypothesized moderation model in Figure 1). In this study, the primary outcome was defined as receipt of at least one new needed service a year after completing the intervention. Because the full ASSIST program is designed to explicitly target advocacy ability, we expected that families taking ASSIST (treatment group) who came in with lower advocacy ability would benefit as equally as those who came in with higher advocacy ability. We hypothesized that families receiving written information (comparison group) who came in with stronger advocacy ability would be more likely to have their service needs met a year later than those with lower advocacy ability. Findings from Li et al. (2024) indicated that among parents of autistic youth, empowerment was most strongly associated with engagement in advocacy activities—an important step toward increased access to services. Therefore, we hypothesized that empowerment would be the most predictive indicator of advocacy ability.

**FIGURE 1:**
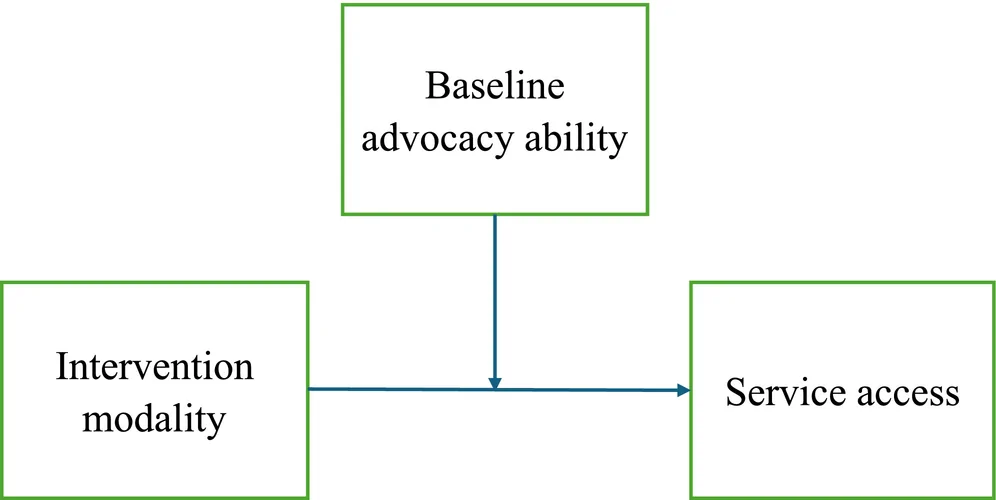
Hypothesized moderation model.

## 2 Methods

### 2.1 Participants

One hundred and eighty‐five participants enrolled in the multi‐site RCT (registered in ClinicalTrials.gov #NCT04173663). The CONSORT diagram can be found in Taylor et al. (2026). Participants were recruited across study sites in three states (IL = 61; TN = 63; WI = 61). Ninety‐one participants were assigned to the treatment group and 94 were assigned to the comparison group. At the 6‐month follow‐up, 163 (88.1%) were retained, and at the 12‐month follow‐up, 157 (84.9%) were retained. Participants in the current study include those who completed interviews at both the 6‐ and 12‐month follow‐up visits (*n* = 149). We excluded participants from the current analysis who did not report any unmet service needs at baseline, resulting in a total of 138 participants who were included in the present study, with *n* = 69 in the treatment group and *n* = 69 in the comparison group. Eligible participants who did not report an unmet service need at baseline (*n* = 11) did not significantly differ in their baseline demographics (all variables reported in Table 1) or indicators of advocacy ability compared to those who reported at least one unmet service need at baseline (*n* = 138; all *p*s > 0.087).

**TABLE 1:** Baseline sample characteristics.

	*n* (%) or *M* (SD)
*Parent characteristics*	
Age	51.44 (6.38)
Gender	
Male	10 (7%)
Female	128 (93%)
Race	
Asian	5 (4%)
Black	13 (9%)
White	113 (82%)
More than one race	3 (2%)
Other	4 (3%)
Latino/a/x	10 (7%)
Highest level of education	
High school degree (or equivalent)	5 (4%)
Some college	18 (13%)
Associate's degree	17 (12%)
Bachelor's degree	47 (34%)
Master's degree	42 (30%)
Ph.D. or professional degree	9 (7%)
Family income	
20,000 or less	1 (1%)
20,001–40,000	14 (10%)
40,001–60,000	17 (12%)
60,001–80,000	18 (13%)
80,001–100,000	14 (10%)
100,001–125,000	16 (12%)
125,001–150,000	12 (9%)
150,001–200,000	16 (12%)
200,001 or higher	18 (13%)
Preferred not to disclose	12 (9%)
*Youth characteristics*	
Age	19.48 (2.71)
Gender	
Male	107 (78%)
Female	29 (21%)
Non‐binary	1 (1%)
I am not sure/I prefer not to respond	1 (1%)
In high school	82 (59%)
Has intellectual disability	49 (36%)
Full scale IQ^a^	87.07 (22.20)
Adaptive behavior^b^	52.30 (21.49)
Co‐occurring mental health condition^c^	87 (63%)

^a^ *n* = 113; measured using two subtests (vocabulary and matrix reasoning) of the Wechsler abbreviated scales of intelligence second edition (Wechsler 2011).

^b^ *n* = 137; measured using the adaptive behavior composite standard score of the vineland adaptive behavior scales third edition (Sparrow et al. 2016).

^c^ Anxiety disorder, panic disorder, bipolar disorder, depression, eating disorder, obsessive compulsive disorder, post traumatic stress disorder, schizophrenia, psychosis, or substance use disorder.

Eligibility criteria for the RCT were as follows: (1) parent or legal guardian of an autistic adolescent/young adult aged 16–26; (2) medical, psychological, or educational documentation of youth's autism diagnosis; (3) youth met a lifetime cut‐off indicating autism on the Social Communication Questionnaire (Rutter et al. 2003); and (4) parents lived in one of the states where the study was conducted (IL, TN, WI) and could attend a 12‐week program at the intervention site. Participants were recruited through autism studies, research registries, disability agencies, school personnel, and autism support groups, as well as targeted emails, mailed letters, phone calls, and word of mouth. Sample characteristics are presented in Table 1.

### 2.2 Procedures

Ethical approval for study procedures was gained through a Single Institutional Review Board (IRB; Vanderbilt University Medical Center IRB of record approval number: 191187). Written consent was obtained from all parents prior to participating in the study. Before receiving the intervention, baseline data were collected from parents either in person or via web conferencing due to restrictions implemented during the COVID‐19 pandemic. After completing baseline data, participants were randomized to the treatment or comparison condition using block randomization within each site, grouped by factors known to impact services: whether the autistic youth was in high school or had exited the school system, and whether the youth had a co‐occurring intellectual disability. Refer to Taylor et al. (2022, 2023) for more details. Parents then participated in their assigned experimental condition, which was delivered in two cohorts at each site. Six and 12 months after the intervention, parents in both conditions participated in follow‐up visits via videoconferencing and an online survey.

### 2.3 Experimental Conditions

Individuals in the treatment condition participated in ASSIST, which is a group‐based 12‐week program led by a facilitator who was a member of a local disability organization with experience in adult services and leading group sessions. Each week's session lasted 2 h and focused on a different topic, including person‐centered planning, models of decision making, supplemental security income, social security disability insurance, health insurance, Medicaid waiver, employment, post‐secondary education, housing, special needs trusts, and advocacy. The sessions began with standardized nationally relevant information and then focused on local information based on participants' geographic location. A set of learning objectives that were common across all sites was used to guide locally relevant material that was presented by a local guest speaker who had expertise in the specific topic for that session. All sessions allowed for group‐based discussion and questions. For each session, participants were given written materials with key points, as well as nationally and locally relevant resources.

Individuals in the comparison group received identical written information on the same schedule as those who took ASSIST but without access to the explanation of the materials or group discussions with facilitators, local experts, and other parent participants. More detailed information on the ASSIST program and RCT can be found in Taylor et al. (2022, 2023).

### 2.4 Measures

#### 2.4.1 Service Needs

At baseline (prior to any intervention), as well as the 6‐ and 12‐month follow‐up visits, parents were asked via a structured interview whether their youth was receiving 21 different services (e.g., speech‐language services, mental health services, social work, respite care; see Lee et al. 2022 for a full list of services) taken from the National Longitudinal Study‐2 (Newman et al. 2011). If their youth was not receiving a service, parents were asked if it was currently needed. From this interview, we determined which services were unmet needs at baseline (i.e., noted as not currently receiving but needed) and then coded whether the youth was receiving any of these services at the 6‐ or 12‐month follow‐up (0 = not receiving any services at follow‐ups that were unmet needs at baseline; 1 = receiving at least one service at follow‐ups that was an unmet need at baseline). We chose to combine the 6‐ and 12‐month follow‐up variables to help mitigate issues with service access due to the COVID‐19 pandemic (Baweja et al. 2022; Burke et al. 2022). Because data collection occurred at different times and pandemic‐related restrictions differed across states, the impact of service access at each follow‐up varied across the sample. Thus, aggregating across both time points reduces the variance that would be associated with restrictions due to the pandemic. In the analysis, the number of services and unmet needs at baseline were included as control variables, which were calculated by summing the number of services parents reported their youth received and the number of unmet needs at that time.

#### 2.4.2 Indicators of Parent Advocacy Ability

All indicators of parent advocacy ability were collected via survey at baseline, prior to intervention.

##### 2.4.2.1 Knowledge of Disability Services

Knowledge of the adult disability service system was measured using a 22‐item multiple‐choice test that asked about facts regarding the service system (Taylor et al. 2023). The scale was created based on instruments used in our team's related research on improving access to services through advocacy (Burke, Goldman, et al. 2016; Taylor et al. 2017). The test included questions such as “Which of the following automatically qualifies your child for Medicare?”, “How can an individual be eligible for a housing voucher?”, and “What does substantial gainful activity mean?” Responses were coded as correct (1) or incorrect (0) with a possible range of scores from 0 to 22.

##### 2.4.2.2 Perceived Advocacy Skills

Parents' perceived advocacy skills were measured using a 10‐item survey used in our previous work (Burke, Goldman, et al. 2016; Moser et al. 2025; Taylor et al. 2017, 2023). The survey asked about parents' perceptions of their advocacy skills using a 5‐point Likert scale, ranging from 1 (“not at all”) to 5 (“excellent”). Items included statements such as “How able are you to effectively communicate with providers/agencies/professionals?” and “How able are you to apply your rights and knowledge of the laws in navigating the adult service system?”. Overall scores were calculated by averaging all items, with a possible range of 1 to 5. Higher scores reflect better perceived advocacy skills. Cronbach's alpha was 0.89 in this sample.

##### 2.4.2.3 Empowerment

Feelings of empowerment were indexed via the Family Empowerment Scale (FES; Koren et al. 1992), which specifically probes for the extent to which parents feel empowered across three contexts: the family, the service system, and the larger community and political environment. The FES was originally developed with families of school‐aged children, so items were adjusted slightly to be relevant for adolescents and young adults (Taylor et al. 2017, 2023). The FES consists of 34 items rated on a 5‐point Likert scale from 1 (“not at all true”) to 5 (“very true”). Items are summed to create an overall score with higher scores indicating greater feelings of empowerment; possible scores range from 34 to 170. Cronbach's alpha was 0.93 in this sample.

### 2.5 Data Analysis

All statistical analyses were conducted using R (R Core Team 2023). To test our research aim, examining whether the impact of intervention modality on receipt of at least one needed service was moderated by baseline advocacy ability, three logistic regressions were conducted—one for each indicator of advocacy ability. The independent variables of interest included the indicator of parent advocacy ability (i.e., knowledge, perceived advocacy skills, or empowerment) at baseline, intervention delivery modality (i.e., ASSIST vs. written materials), and the interaction between advocacy ability and intervention delivery modality. The dependent variable for each model was whether or not youth received at least one service at the 6‐ or 12‐month follow‐up that was an unmet need at baseline. Study site (Illinois, Tennessee, Wisconsin), cohort, whether the youth was in high school at baseline, parent educational attainment, as well as the number of unmet service needs and number of services at baseline were considered as potential control variables for each model. Study site and high school status were considered, given that access to services can differ across states (e.g., spending, eligibility, benefits) and between those in and out of high school (Laxman et al. 2019; Shattuck et al. 2011). We considered controlling for parents' educational attainment, as this can also impact service access (Magaña et al. 2013; Pickard and Ingersoll 2016). The number of services youth received was considered to account for those already integrated into the service system, and the number of unmet needs at baseline was considered, as individuals with more unmet needs have a greater opportunity to have a service need met. Finally, cohort was considered as a control variable to account for possible differences in the timing and implementation of the intervention across cohorts.

Given that we had limited power to detect statistically significant interactions, we employed a model‐building approach, starting with the simplest model (i.e., the variables of interest) and including covariates only when they improved model fit, as indicated by a lower Akaike Information Criterion (AIC). This approach allowed us to preserve as much power as possible to detect interactions. We found that across all models, the inclusion of the number of unmet service needs and number of services at baseline as control variables resulted in the best‐fitting model (lowest AIC). All variables were grand‐mean centered.

When a significant interaction was detected, we conducted two follow‐up tests to further interpret the effect. First, simple slopes analyses were used to evaluate the effect of baseline advocacy ability on the likelihood of having a service need met for each intervention modality. Additionally, a Johnson‐Neyman (J‐N) analysis was used to identify the region of baseline advocacy ability where the intervention modalities significantly differed in their predicted probabilities of having a service need met.

## 3 Results

### 3.1 Baseline Knowledge of Adult Disability Services

Table 2 presents the estimates from the logistic regression model testing the effect of intervention modality, knowledge of disability services at baseline, and their interaction on having at least one service need met over the course of a year. Neither the main effects nor the interaction between knowledge and intervention modality was statistically significant.

**TABLE 2:** Estimates from the logistic regression testing the interaction between baseline knowledge and intervention modality predicting whether youth had at least one service need met.

	Estimate	SE	*p*	Adjusted OR
Intercept	0.45	0.19	0.020	
Number of unmet service needs at baseline	0.36	0.10	< 0.001	1.43
Number of services at baseline	0.16	0.07	0.023	1.17
Intervention modality	−0.24	0.37	0.515	0.78
Baseline knowledge	−0.01	0.06	0.870	0.99
Baseline knowledge × intervention modality	−0.09	0.11	0.421	0.91

### 3.2 Perceived Advocacy Skills at Baseline

Table 3 shows the estimates from the logistic regression testing the effect of intervention modality, perceived advocacy skills at baseline, and their interaction on having at least one service need met over the course of a year. There was no main effect of perceived advocacy skills or intervention modality; however, a statistically significant interaction was detected between intervention modality and perceived advocacy skills. A simple slopes analysis showed that the effect of perceived advocacy skills at baseline was stronger for participants who took ASSIST, but this association was not significant (*b* = 0.71, *p* = 0.069). No statistically significant association was detected for participants who received written materials (*b* = −0.60, *p* = 0.142). A J‐N analysis indicated that participants who took ASSIST had a significantly higher likelihood of getting a service need met than those who received written materials when baseline levels of perceived advocacy skills exceeded 0.53 (*p* < 0.05), which corresponds to a value of 4.07 (uncentered); 22% of the sample had scores that fell above this threshold. The J‐N region of significance and estimated slopes stratified by intervention modality are shown in Figure 2.

**TABLE 3:** Estimates from the logistic regression testing the interaction between perceived advocacy skills at baseline and intervention modality predicting whether youth had at least one service need met.

	Estimate	SE	*p*	Adjusted OR
Intercept	0.45	0.19	0.019	
Number of unmet service needs at baseline	0.35	0.09	< 0.001	1.42
Number of services at baseline	0.16	0.07	0.025	1.18
Intervention modality	−0.28	0.38	0.467	0.76
Baseline perceived advocacy skills	0.05	0.28	0.857	1.05
Baseline perceived advocacy skills × intervention modality	−1.31	0.56	0.020	0.27

**FIGURE 2:**
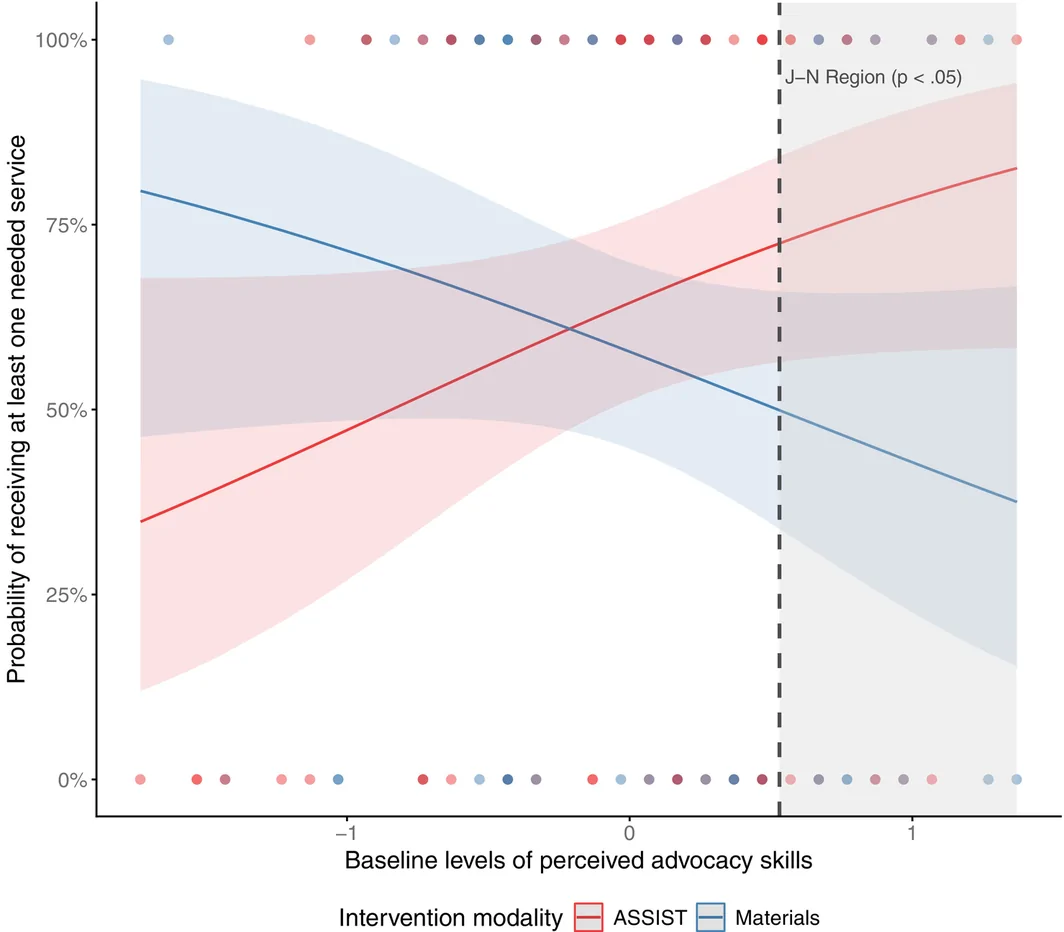
Baseline levels of perceived advocacy skills predicting receipt of needed services by intervention modality. For ease of interpretation, we plot predicted probabilities for each intervention modality.

### 3.3 Baseline Empowerment

Table 4 presents the estimates from the logistic regression model testing the effect of intervention modality, baseline empowerment levels, and their interaction on having at least one service need met over the course of a year. No main effect of baseline empowerment or intervention modality was detected. There was a statistically significant interaction between empowerment and intervention modality. A simple slopes analysis indicated that participants who took ASSIST were more likely to have at least one service need met if they had higher levels of empowerment prior to the intervention (*b* = 0.03, *p* = 0.028). There was no statistically significant association between empowerment and having a service need met for those who were given written materials (*b* = −0.02, *p* = 0.322). A J‐N analysis indicated that participants who took ASSIST had a significantly higher likelihood of getting a service need met than those who received written materials when baseline levels of empowerment exceeded 13.42 (*p* < 0.05), corresponding to a value of 138.11 (uncentered) on the FES; 23% of the sample had scores that fell above this threshold. The J‐N region of significance and estimated slopes stratified by intervention modality are shown in Figure 3.

**TABLE 4:** Estimates from the logistic regression testing the interaction between baseline empowerment levels and intervention modality predicting whether youth had at least one service need met.

	Estimate	SE	*p*	Adjusted OR
Intercept	0.50	0.20	0.012	
Number of unmet service needs at baseline	0.38	0.10	< 0.001	1.46
Number of services at baseline	0.17	0.08	0.026	1.19
Intervention modality	−0.31	0.39	0.420	0.73
Baseline empowerment levels	0.01	0.01	0.507	1.01
Baseline empowerment levels × intervention modality	−0.05	0.02	0.028	0.95

**FIGURE 3:**
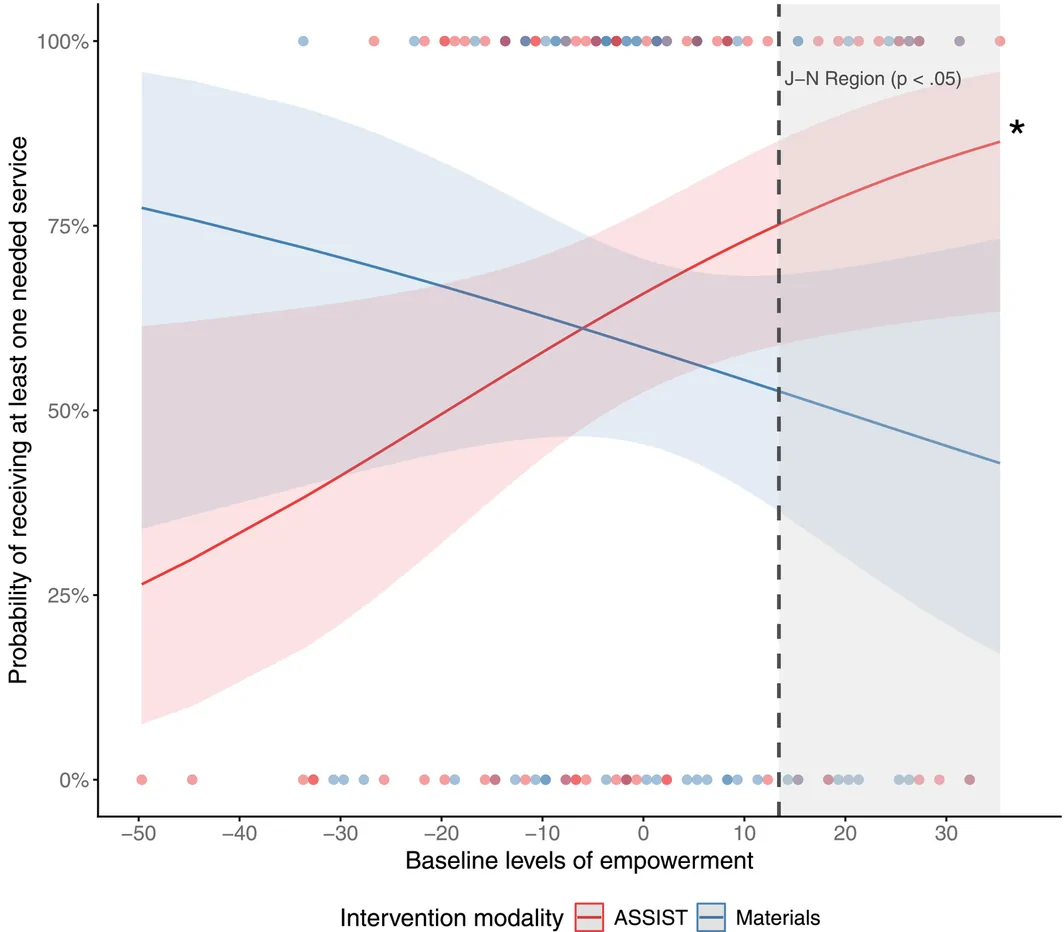
Baseline levels of empowerment predicting receipt of needed services by intervention modality. For ease of interpretation, we plot predicted probabilities for each intervention modality. *p* < 0.05.

## 4 Discussion

The present study investigated whether baseline levels of parent advocacy ability (i.e., knowledge, perceived advocacy skills, and empowerment) moderated the effect of treatment on service access for parents receiving the ASSIST program versus those receiving written materials. We found that parents with higher baseline levels of empowerment were more likely to have their youth's service needs met a year later, but this effect was only observed for those who participated in the ASSIST program. Results also showed that parents taking ASSIST who reported high levels of perceived advocacy skills and empowerment at baseline were more likely to have a service need met compared to those who received written information, whereas there was no effect of intervention modality at lower levels of perceived advocacy skills or empowerment. These findings have important implications for identifying which families are most likely to benefit from a parent advocacy program versus written information and highlight perceived advocacy skills and empowerment as key factors in securing services for autistic youth.

The positive relationship between empowerment and service access for the treatment group aligns with prior research linking higher levels of empowerment with increased satisfaction and involvement with services (Curtis and Singh 1996; Resendez et al. 2000). It also mirrors findings from studies focused on self‐efficacy, a related construct, and its relation to treatment outcomes (e.g., Clark and Dodge 1999; Hayward and Stynes 2021; Jakubovski and Bloch 2016; Náfrádi et al. 2017). These studies demonstrate that individuals with high baseline self‐efficacy benefit most from treatment, while those with low initial levels of self‐efficacy benefit the least. Though this pattern of findings is common, existing research indicates empowerment can be increased in parents of autistic children (Magaña et al. 2017), which suggests it is a malleable construct that may need to be targeted more explicitly in interventions.

For families who had high levels of empowerment and perceived advocacy skills, ASSIST was particularly effective in translating information into service access compared to parents who received written materials. That is, parents who took ASSIST and reported high baseline levels of empowerment (score exceeding 136.32 on a scale of 34–170) and perceived advocacy skills (score exceeding 4 on a scale of 1–5) were significantly more likely to have a service need met a year later compared to those who received written materials. Approximately one‐fourth of the sample reported baseline advocacy skills and empowerment levels above these thresholds, indicating a considerable number of parents could leverage their existing advocacy ability to improve service outcomes with ASSIST. On the other hand, a large portion (three‐quarters of the sample) may have benefitted from more targeted or intensive advocacy interventions. There was no difference between intervention modalities in the likelihood of having a service need met among families with lower levels of empowerment or perceived advocacy skills, suggesting that ASSIST was not more effective than written materials for these participants.

These findings contrast with our previous study, which demonstrated that participants entering ASSIST with lower baseline advocacy ability made the greatest subsequent gains (Taylor et al. 2023). However, rather than being incongruent, these findings may point toward the utility of a stepped approach for advocacy interventions. It could be that to see changes in getting service needs met—which can take time (Burke et al. 2018)—participants need to come in feeling empowered and ready to advocate. Parents who feel less empowered may benefit from an additional empowerment‐focused intervention delivered prior to ASSIST to ensure they can fully capitalize on the program and ultimately improve service access for their youth. Thus, a stepped‐care adaptive intervention approach could be considered, in which advocacy ability is targeted first, and ASSIST is introduced once advocacy ability has reached a certain threshold. Future studies should explore this strategy as a way to level the playing field for families with lower advocacy ability.

Although no significant association emerged between knowledge and service access for either intervention modality, future research should explore potential interactions among indicators of advocacy ability. Understanding how different profiles (e.g., parents with high empowerment coupled with low knowledge versus parents with high knowledge who have low empowerment levels) shape treatment outcomes can inform more personalized interventions. Another important consideration is determining whether certain components of advocacy ability should be targeted before others. Given that empowerment has been shown to be a mediator through which knowledge impacts participation in advocacy activities in this sample (Li et al. 2024) and in a sample of parents of children with intellectual and developmental disabilities (Burke, Li, and Rossetti 2025), it may be that knowledge is necessary but not sufficient. In other words, knowledge could be an important precursor to understanding how to access services, but empowerment and perceived advocacy skills are needed to succeed in receiving those services. While qualitative work highlights the fact that knowledge is only one hurdle and persistence in advocacy and empowerment are critical for getting service needs met (Ryan and Quinlan 2018; Smith‐Young et al. 2022), rigorous longitudinal research is needed to clarify how these indicators of advocacy ability interact to facilitate service access and how they can be strengthened through parent advocacy programs.

This study has several limitations and areas of consideration for future work. First, our sample is highly educated and mostly White. The education level of this sample, with 71% holding a bachelor's or graduate degree, could have inflated the comparison group's success rate in obtaining needed services, as participants with higher educational attainment may be better able to use written information to access services. Future studies with greater racial and ethnic diversity are also needed to understand the ways in which baseline treatment targets may impact intervention effects of parent advocacy programs. ASSIST has been adapted for Spanish‐speaking families, and preliminary findings comparing White and Latino caregivers indicate differences in baseline levels of treatment targets; Latino families reported higher levels of family empowerment but lower levels of knowledge and perceived advocacy skills (Burke, Ramos‐Torres, et al. 2025). These findings underscore the importance of examining how individual differences in advocacy ability may influence intervention outcomes across different racial and ethnic groups—particularly given that prior research has documented inequitable access to services and lack of representation in intervention studies for minoritized racial and ethnic groups (Steinbrenner et al. 2022). Secondly, although we observed high rates of met service needs for participants across both intervention modalities, it remains unclear who benefits most from written materials only. Alternative research methods, such as a Sequential Multiple Assignment Randomized Trial (SMART; Collins et al. 2007) design, could clarify which individuals respond to comprehensive written materials and who may need additional support. This type of design could inform a multi‐tiered system of support (MTSS; Stoiber and Gettinger 2015), in which more intensive resources are targeted to those who need them.

Additional limitations include our relatively small sample size for testing moderation effects; thus, there may have been meaningful associations that we were not able to detect in this study, and greater emphasis should be placed on effect sizes when interpreting the results. Also, data collection occurred during the COVID‐19 pandemic, which disrupted services (Baweja et al. 2022; Burke et al. 2022). Although examining service access at two timepoints over the course of a year likely mitigated some of this impact, it is still an important factor to consider when interpreting findings. Moreover, because we collapsed two timepoints of data, we were unable to examine temporal effects, such as whether ASSIST accelerated the receipt of needed services, which is an important direction for future work. Additionally, although the FES demonstrated strong internal consistency in our sample, it was originally developed for school‐aged children; thus, its adaptation and use in an older sample may limit its validity. Further research is needed to validate these adaptations in a larger sample. Finally, we did not include an untreated group; therefore, it is not possible to determine the effect of the ASSIST intervention on getting a service need met compared to information readily available in the community.

Despite these limitations, this study contributes to the literature by exploring strategies for delivering information about advocacy and the adult disability service system to families and identifying the conditions under which a parent advocacy program is the most effective. One strength of the study is our granular approach to measuring advocacy, separating it into three distinct components, which allowed us to detect associations that may have been masked using an overall advocacy ability measure. Other key strengths include controlling for the number of services youth were receiving and unmet needs at baseline and using needed services as our outcome variable, as a count of received services can be confounded with functional needs. For instance, an autistic adult with co‐occurring intellectual disability may rely on more services than someone without intellectual disability but may still have a critical service need that is not met. An autistic adult without intellectual disability who is receiving fewer services may also have an important unmet service need. Thus, the total number of services received does not necessarily reflect whether service needs are being addressed. By examining whether a needed service was received, our outcome measure was tailored to each individual's needs, helping to level the playing field (Burke et al. 2024; Burke and Taylor 2023). In sum, our findings underscore the importance of considering baseline levels of advocacy ability as a way to inform more personalized approaches to service access through parent advocacy programs.

## Funding

This work was supported by the National Institute of Mental Health (R01 MH116058), National Institute of Child Health and Human Development (P50 HD103537 and P50 HD105353), National Center for Advancing Translational Sciences (UL1 TR000445), and the Administration on Community Living (90DDUC0153).

## Conflicts of Interest

The authors declare no conflicts of interest.

## Data Availability

The data that support the findings of this study are openly available in the National Institute of Mental Health (NIMH) Data Archive at https://nda.nih.gov/ in accordance with NIMH guidelines.
